# Inactivation of Group 1B Phospholipase A_2_ Enhances Disease Recovery and Reduces Experimental Colitis in Mice

**DOI:** 10.3390/ijms242216155

**Published:** 2023-11-10

**Authors:** April M. Haller, Patrick R. Wolfkiel, Anja Jaeschke, David Y. Hui

**Affiliations:** 1 Department of Pathology, University of Cincinnati College of Medicine, Cincinnati, OH 45237, USA; cannonam@ucmail.uc.edu (A.M.H.); jaeschaa@ucmail.uc.edu (A.J.); 2 Molecular Genetics, Biochemistry and Microbiology Graduate Program, University of Cincinnati, Cincinnati, OH 45267, USA; patwolfki@gmail.com

**Keywords:** phospholipase A_2_, inflammatory bowel disease, myeloid inflammatory response, intestinal stem cells

## Abstract

Phospholipase A_2_ (PLA_2_) enzymes influence inflammatory bowel disease in both positive and negative manners depending on the type of PLA_2_ that is expressed. This study explored the influence of the abundantly expressed Group 1B PLA_2_ (PLA2G1B) on ulcerative colitis. Wild-type C57BL/6J mice and *Pla2g1b^−/−^* mice were treated with dextran sulfate sodium (DSS) for 5 days to induce epithelial injury, followed by another 5 days without DSS for recovery. The *Pla2g1b^−/−^* mice displayed significantly less body weight loss, colitis pathology, and disease activity indexes compared to the wild-type mice. The differences in colitis were not due to differences in the colonic lysophospholipid levels, but higher numbers of stem and progenitor cells were found in the intestines of *Pla2g1b^−/−^* mice compared to the wild-type mice. The DSS-treated *Pla2g1b^−/−^* mice also showed higher expressions of genes that are responsible for epithelial repair and lower expressions of proinflammatory cytokine genes in the colon, as well as reduced inflammatory cytokine levels in the plasma. In vitro experiments revealed the PLA2G1B stimulation of inflammatory cytokine expression by myeloid cells. PLA2G1B inactivation protects against DSS-induced colitis in mice by increasing the intestinal stem cell reservoir for epithelial repair and reducing myeloid cell inflammation in the diseased colon. Thus, PLA2G1B may be a target for colitis management.

## 1. Introduction

Ulcerative colitis is a chronic inflammatory disease of the gastrointestinal tract with unknown etiology. Pathologically, it is characterized by colonic mucosal injury and immune cell infiltration to exacerbate tissue inflammation and crypt destruction. Major risk factors for ulcerative colitis include genetic predisposition, childhood environment, stress, intestinal dysbiosis, and the chronic consumption of a high-fat diet [1,2,3,4]. Several studies indicated that differences in the lipid and amino acid metabolisms in the digestive tract may be key determinants of inflammatory bowel disease [5,6]. In particular, the lipidomic profiling of intestinal and colonic mucosa revealed significantly lower levels of phosphatidylcholine (PC) and the corresponding increase in the lysophosphatidylcholine (LPC) levels in colitis patients compared to the control subjects [6,7]. These observations led to the hypothesis that enzymes that are responsible for PC hydrolysis to LPC may be involved in the pathogenesis of ulcerative colitis [7].

Enzymes that hydrolyze PC to LPC are members of the phospholipase A_2_ (PLA_2_) superfamily that includes six major classes based on their cellular locations. These include the secretory PLA_2_ enzymes, which hydrolyze extracellular phospholipids to lysophospholipids, as well as intracellular enzymes such as the cytosolic PLA_2_ and the calcium-independent PLA_2_, which hydrolyze phospholipids in different intracellular compartments [8]. The products of PLA_2_ reactions, lysophospholipids, and nonesterified fatty acids, are bioactive lipid metabolites that serve as intracellular and extracellular signals in regulating cell functions and pathophysiological process such as obesity/diabetes, cardiovascular disease, cancer, neurodegenerative disorders, and inflammatory bowel disease [9]. These lipid metabolites may also serve as substrates for the synthesis of other active lipid metabolites such as prostaglandins, thromboxane, leukotrienes, and epoxyeicosatrienoic acids that modulate cardiometabolic and inflammatory disease pathogenesis [10].

Several PLA_2_ enzymes have been shown to influence inflammatory diseases in the gastrointestinal tract. Interestingly, various PLA_2_ enzymes appeared to influence inflammatory bowel disease in opposing manners depending on the cell type and where the enzyme is expressed. For example, the calcium-independent Group VIA PLA_2_, which is expressed intracellularly in the colon, was shown to protect against inflammatory bowel disease, and its deletion in mice increased the susceptibility to chemical-induced colitis [11]. In contrast, the cytosolic Group IVA PLA_2_ increases colonic inflammation, and its inactivation is beneficial in suppressing inflammatory bowel disease [12,13]. Likewise, whereas the secretory Group III PLA_2_ is proinflammatory and its inactivation protects mice against chemical-induced colitis [14], the Group X secretory PLA_2_ (PLA2G10) expressed in non-hematopoietic but not hematopoietic cells is anti-inflammatory and also protects against colitis [15,16]. The secretory Group IIA PLA_2_ (PLA2G2A), which is absent in C57BL/6 mice, the most commonly used mouse model to study disease pathogenesis, was also found to be proinflammatory and promotes colitis when overexpressed in C57BL/6 mice [16]. Moreover, the selective inhibition of PLA2G2A was also found to reduce colitis in rats [17]. The lack of this enzyme in *Pla2g10*-null mice displayed improved inflammatory responses compared to *Pla2g10*-null mice with PLA2G10A expression [16].

One of the most abundant PLA_2_ enzymes in the digestive tract is the Group 1B PLA_2_ (PLA2G1B), which is secreted by the pancreas into the intestinal lumen in response to food intake. We have previously shown that PLA2G1B hydrolyzes phospholipids to lysophospholipids during food digestion to aid in lipid absorption, and the resulting lysophospholipids are transported to the liver to reduce fatty acid oxidation and cause mitochondria dysfunction, thereby promoting hyperlipidemia, obesity/diabetes, and atherosclerosis [18,19,20,21,22]. Interestingly, despite its role in metabolic disease pathogenesis and despite the fact that its inactivation is protective against diet-induced cardiometabolic diseases [23], PLA2G1B that is expressed in the pancreas and intestinal epithelial cells also has health benefits, including its anthelmintic properties that protect the gut epithelium from repeated helminth infection after initial anthelmintic treatment [24]. Its anthelmintic property is unrelated to the adaptive and innate immunity of the host, but it is due to PLA2G1B’s killing of the larvae by hydrolyzing their membrane phospholipids. Whether PLA2G1B enhances or protects against mucosal injury and inflammation in intestinal bowel disease has not been explored previously. This study compares wild-type and *Pla2g1b^−/−^* mice in response to dextran sulfate sodium (DSS)-induced colitis to address this issue.

## 2. Results

### 2.1. Genetic Inactivation of PLA2G1B Protects against DSS-Induced Colitis

**Figure 1:**
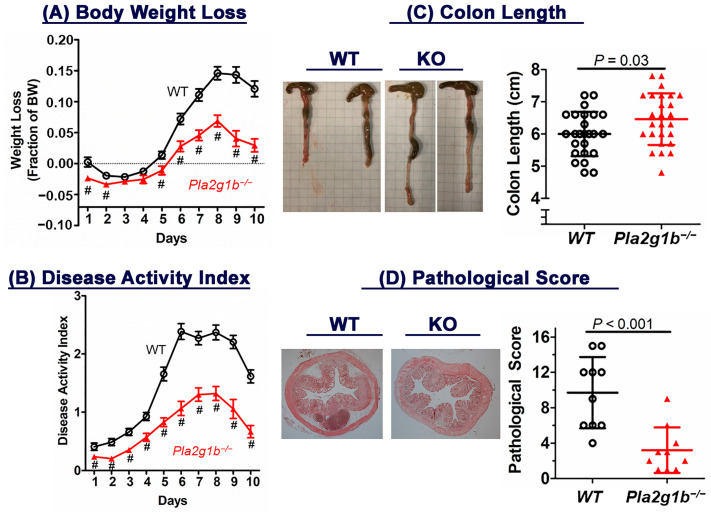
Inactivation of PLA2G1B reduces DSS-induced colitis in mice. Drinking water supplemented with 3% DSS was administered to 12-week-old male wild-type C57BL/6J (WT, N = 27) and *Pla2g1b^−/−^* (N = 26) mice for 5 days followed by water without DSS for 5 additional days. (**A**) Body weight loss and (**B**) disease activity index were monitored over a 10-day period. Data = mean ± SEM; # *p* < 0.01 versus WT group. (**C**) Colon lengths (each block = 0.6 cm) in WT and *Pla2g1b^−/−^* mice as well as (**D**) pathological scores in WT (N = 10) and *Pla2g1b^−/−^* (N = 10) mice were assessed at the end of the 10-day period. Statistical significance was evaluated using Student’s *t*-test with the *p* values as indicated.

The role of PLA2G1B in inflammatory bowel disease was assessed by comparing wild-type C57BL/6J and *Pla2g1b^−/−^* mice in response to colonic epithelial cell injury induced by 5 days of oral DSS treatment [25]. The results showed minimal body weight loss in both groups during the initial 5-day period, although the differences between the wild-type and *Pla2g1b^−/−^* mice were significant (Figure 1A). Interestingly, while the wild-type mice continued to lose weight during the 5-day recovery period, the body weights of the *Pla2g1b^−/−^* mice stabilized during the recovery period, and they appeared to regain weight toward the end of the 10-day study period (Figure 1A). An analysis of the disease activity index (DAI) score revealed evidence of colitis in both groups, but the disease in the *Pla2g1b^−/−^* mice was significantly milder compared to that observed in the wild-type mice through the initial 5-day injury period as well as 5 days thereafter during the recovery period (Figure 1B). At the end of the study period, the colon lengths in the wild-type mice were significantly shorter than those observed in the *Pla2g1b^−/−^* mice (Figure 1C). A histological characterization of the colon revealed significantly less damage of the crypts, including a mostly preserved crypt architecture in the *Pla2g1b^−/−^* mice (Figure 1D).

### 2.2. Resistance of Pla2g1b^−/−^ Mice to DSS-Induced Colitis Is Unrelated to Lysophospholipid Content in Colon

**Figure 2:**
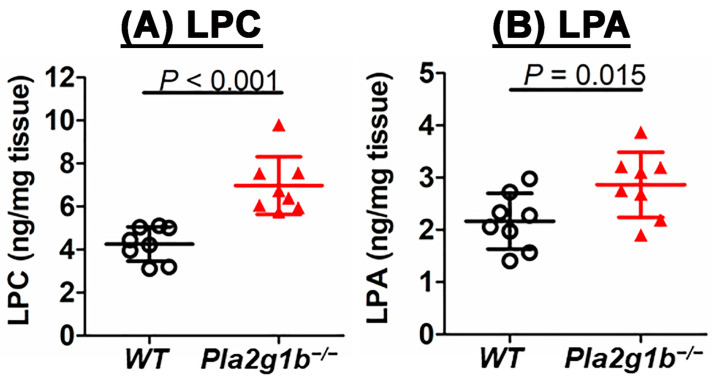
Colonic contents of (**A**) LPC and (**B**) LPA in wild-type (WT, N = 8) and *Pla2g1b^−/−^* (N = 8) mice after DSS-induced injury. Phospholipids and lysophospholipids were extracted from colons for analysis. Data are shown as mean ± SD with *p* values evaluated using Student’s *t*-test, as indicated.

Our previous studies have shown that the resistance of *Pla2g1b^−/−^* mice to diet-induced obesity and hyperglycemia is due to reduced lysophospholipid levels in the intestinal lumen and their transport to the liver [18,19]. In view of the relationship between LPC levels and colitis in humans [6,7], we measured the LPC and lysophosphatidic acid (LPA) levels in the colons of wild-type and *Pla2g1b^−/−^* mice after the DSS treatment. In contrast to the lower LPC and LPA levels in the intestinal lumens of the *Pla2g1b^−/−^* mice under normal conditions [18,19], higher LPC and LPA levels were observed in the colons of the DSS-treated *Pla2g1b^−/−^* mice compared to the wild-type mice (Figure 2). These observations indicated that the PLA2G1B-catalyzed phospholipid conversion to lysophospholipid in the intestine plays a minimal role in phospholipid–lysophospholipid homeostasis in the colon. Moreover, the resistance of *Pla2g1b^−/−^* mice to DSS-induced colitis despite the elevated colonic LPC and LPA levels suggested that an increased lysophospholipid content in the colon is not sufficient for colitis pathogenesis.

### 2.3. PLA2G1B Inactivation Increases Intestinal Stem Cells

**Figure 3:**
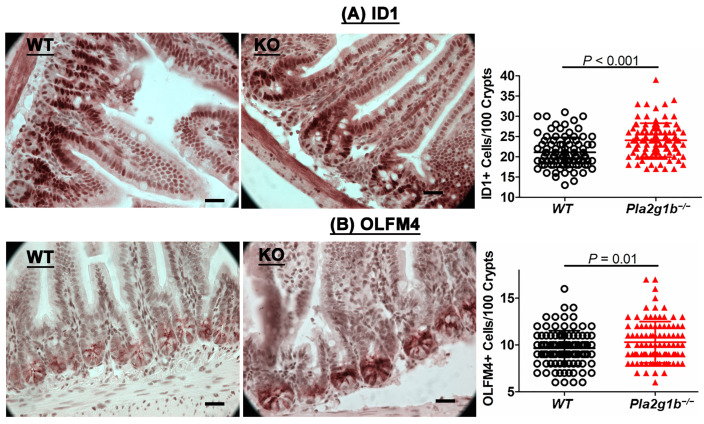
PLA2G1B inactivation increased stem and progenitor cell population in the intestine. Intestines of wild-type (WT, N = 8) and *Pla2g1b^−/−^* (N = 8) mice were immunostained with antibodies against Id1 (**A**) or Olfm4 (**B**) to identify intestinal stem and progenitor cells. Representative images (scale bar = 100 µm) and mean ± SD of data for stem cells per 100 crypts are shown. Differences were evaluated using Student’s *t*-test with *p* values, as indicated.

Previous studies have shown that PLA2G2A and PLA2G10 are normally expressed intracellularly in Paneth and Paneth/goblet-like cells along the digestive tract to inhibit intestinal stem cell function and differentiation for the maintenance of cell homeostasis. However, these enzymes can be secreted upon inflammation to inhibit intestinal stem cell growth [16]. Importantly, the inactivation of PLA2G10 in PLA2G2A-defective C57BL/6J mice was found to promote cell growth and improve recovery from inflammation to limit DSS-induced colitis [16]. Since PLA2G1B is an enzyme that is secreted into the intestinal lumen, we examined the intestines of wild-type and *Pla2g1b^−/−^* mice histologically and found increased numbers of Id1- and olfactomedin 4 (Olfm4)-positive intestinal stem and progenitor cells in the *Pla2g1b^−/−^* mice compared to the wild-type mice (Figure 3). The increased reservoir of intestinal stem and progenitor cells in the *Pla2g1b^−/−^* mice may be one factor that is responsible for the improved recovery of the intestinal epithelium and reduced colitis observed in these animals.

### 2.4. PLA2G1B Inactivation Increases Expression of Genes That Promote Epithelial Repair

**Figure 4:**
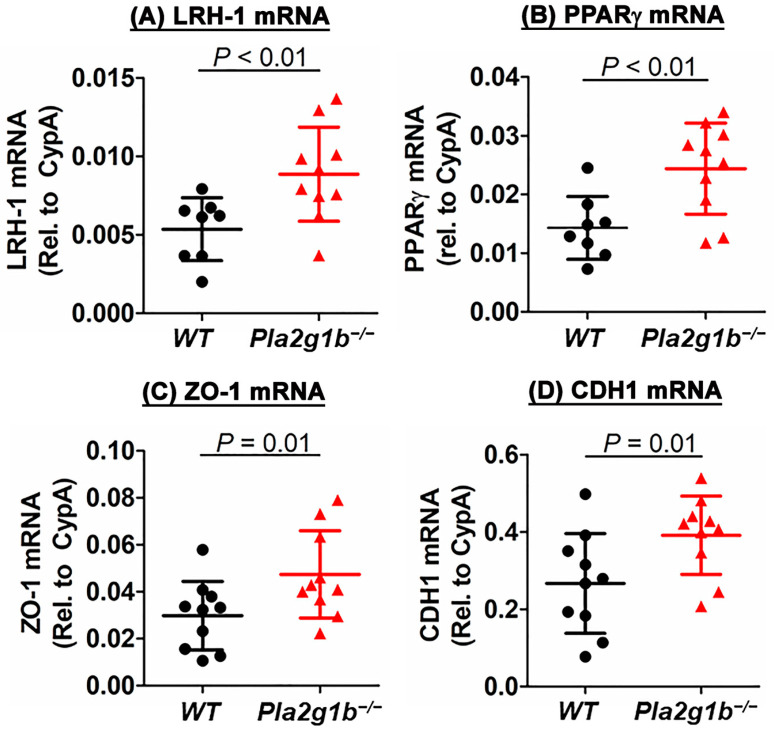
PLA2G1B inactivation promotes expression of epithelial repair genes. Total RNA was prepared from the colons of DSS-treated wild-type (N = 8) and *Pla2g1b^−/−^* (N = 10) mice. RT-PCR was performed to assess expression levels of (**A**) LRH-1, (**B**) PPARγ, (**C**) ZO-1, and (**D**) CDH1 using cyclophilin (CypA) as the control. Data represent mean ± SD with *p* values indicated as determined using Student’s *t*-test.

Our data showing the improved recovery of DSS-induced colitis along with reduced colonic crypt damage and increased numbers of stem and progenitor cells in the intestines of *Pla2g1b^−/−^* mice compared to the wild-type mice suggest that PLA2G1B inactivation may be related to increased epithelial repair. To test this possibility, RNA was prepared from the colons of DSS-treated wild-type and *Pla2g1b^−/−^* mice to assess the expression of genes related to epithelial proliferation and repair. The results showed that the expression levels of liver receptor homolog-1 (LRH-1) was significantly higher in the DSS-treated *Pla2g1b^−/−^* mice compared to the wild-type mice (Figure 4A). LRH-1 is a nuclear receptor that has been previously shown to promote intestinal crypt cell renewal, and its reduced expression in the colon was correlated with inflammatory bowel disease in humans [26,27]. The mechanism underlying LRH-1 protection against colitis is due to its promotion of glucocorticoid synthesis [27], which leads to the increased expression of peroxisome proliferator-activated receptor-γ (PPARγ) and the downstream effects of PPARγ in the protection against colitis and inflammatory bowel disease [28,29,30,31]. Indeed, the PPARγ expression levels were also found to be higher in the colons of the *Pla2g1b^−/−^* mice compared to the wild-type mice (Figure 4B). Additionally, the tight junction proteins, Zonula Occludens 1 (ZO-1) and E-cadherin (CDH1), that are important for epithelial integrity maintenance [32,33] were also expressed at a higher levels in the colons of the DSS-treated *Pla2g1b^−/−^* mice compared to the wild-type mice (Figure 4C,D). Taken together, these results are consistent with the interpretation that PLA2G1B inactivation protects against DSS-induced colitis by accelerating epithelial repair.

### 2.5. PLA2G1B Inactivation Reduces DSS-Induced Inflammation

**Figure 5:**
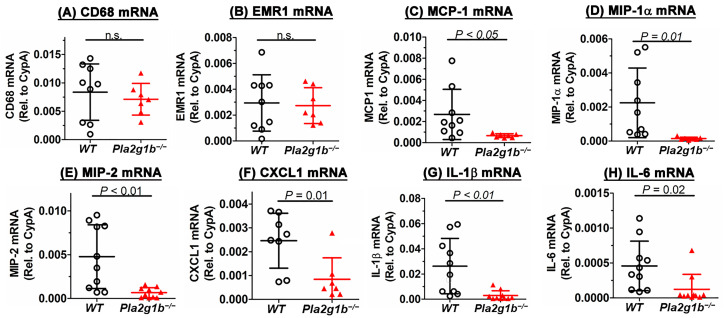
PLA2G1B inactivation reduces inflammatory cytokine expression in colons of DSS-treated mice. Total RNA was prepared from the colons of DSS-treated wild-type (WT, N = 9) and *Pla2g1b^−/−^* mice (N = 7). RT-PCR was performed to assess expression levels of (**A**) CD68, (**B**) EMR1, (**C**) MCP-1, (**D**) MIP-1α, (**E**) MIP-2, (**F**) CXCL1, (**G**) IL-1β, and (**H**) IL-6. The data represent mean ± SD with *p* values determined using Student’s *t*-test, as indicated. n.s. = not significant.

**Figure 6:**
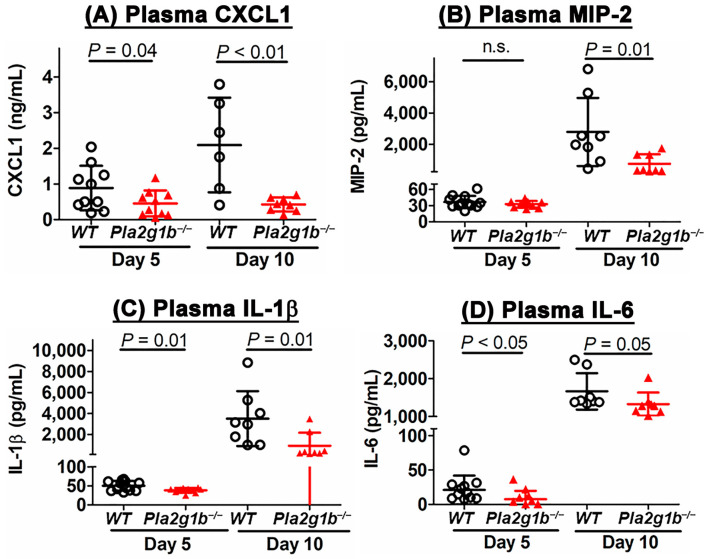
PLA2G1B inactivation lowers inflammatory cytokines in plasma of DSS-treated mice. Wild-type (WT) and *Pla2g1b^−/−^* mice (N = 8–10 in each group) were treated with DSS-supplemented water for 5 days. Plasma was collected at day 5 and day 10 (5 days after DSS treatment) for ELISA determination of (**A**) CXCL1, (**B**) MIP-2, (**C**) IL-1β, and (**D**) IL-6. The data represent mean ± SD with significance evaluated using Student’s *t*-test, as indicated.

Another hallmark of colitis is an acute innate inflammatory response due to neutrophil and macrophage infiltration into the mucosa [25]. In particular, the elevated expression of macrophage inflammatory protein-2 (MIP2, also known as CXCL2) has been shown to correlate positively with disease severity [34]. In view of previous studies showing that PLA2G1B inactivation suppresses inflammation in chronic metabolic diseases [23], we explored whether the reduced colitis observed in the *Pla2g1b^−/−^* mice may also be due to the reduced innate inflammation in the colon. While a quantitative RT-PCR analysis did not reveal a difference in the expressions of macrophage gene markers, CD68 and EMR1, between the colons of the DSS-treated wild-type and *Pla2g1b^−/−^* mice (Figure 5A,B), the expressions of inflammatory cytokines such as CCL2/MCP1, CCL3/MIP-1α, CXCL1/KC, CXCL2/MIP2, IL-1β, and IL-6 were significantly lower in the colons of the DSS-treated *Pla2g1b^−/−^*mice compared to the wild-type mice (Figure 5C–H). Correspondingly, the plasma levels of CXCL1/KC, CXCL2/MIP2, IL-1β, and IL-6 were found to increase progressively in the wild-type mice after the DSS treatment, but their levels in the *Pla2g1b^−/−^* mice remained low throughout the experimental period after the DSS treatment (Figure 6). Taken together, these data indicated that PLA2G1B inactivation did not affect monocyte/macrophage infiltration into the colon in response to DSS-induced epithelial injury, but it reduced inflammatory cytokine production to limit inflammation and colitis.

### 2.6. PLA2G1B Directly Activates Inflammatory Cytokine Production in Myeloid Cells

**Figure 7:**
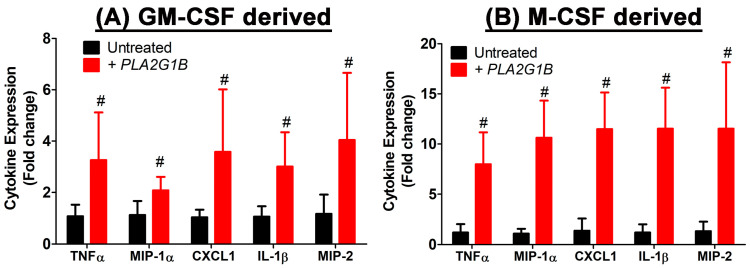
PLA2G1B directly elevates inflammatory cytokine production via myeloid cells. Bone marrow cells isolated from wild-type C57BL/6J mice were incubated with (**A**) GM-CSF or (**B**) M-CSF to induce differentiation into mature myeloid cells. The myeloid cells were incubated with (red bars) or without (black bars) 10 µg/mL PLA2G1B for 3 h prior to RNA isolation. RT-PCR was performed to assess expression of the selected inflammatory cytokine genes as indicated. The # symbol indicates significant difference from wild-type group at *p* < 0.01 (N = 10 biological replicates), as determined using Student’s *t*-test.

The relationship between PLA2G1B and inflammatory cytokine production was explored in vitro by ascertaining the potential of a direct influence of exogenous PLA2G1B, mimicking the influence of PLA2G1B secreted into the intestinal and colonic lumen on cytokine expression by bone marrow-derived myeloid cells. For these experiments, bone marrow was obtained from wild-type C57BL/6J mice and differentiated into mature myeloid cells via incubation with granulocyte-macrophage colony-stimulating factor (GM-CSF) or macrophage colony-stimulating factor (M-CSF). The differentiated myeloid cells were then incubated with or without 10 µg/mL of bovine PLA2G1B for 3 h prior to RNA isolation for the RT-PCR analysis of gene expression. The results showed that exogenously added PLA2G1B elevated the expressions of the inflammatory cytokines, TNFα, MIP-1α, CXCL1, IL-1β, and MIP-2, by ~2–4 fold in the GM-CSF-activated myeloid cells (Figure 7A) and by ~8–12 fold in the M-CSF-activated myeloid cells (Figure 7B). These results suggested that, despite the presence of similar levels of macrophages in the colons of the DSS-treated wild-type and *Pla2g1b^−/−^* mice, significantly lower levels of proinflammatory cytokine expression were detected in the colons of the DSS-treated *Pla2g1b^−/−^* mice compared to the wild-type mice. These results suggest that the higher cytokine expression levels detected in the DSS-treated colons of wild-type mice compared to the *Pla2g1b^−/−^* mice were likely due to exogenous PLA2G1B secreted into the digestive tract, stimulating the macrophage inflammatory response in the wild-type mice.

## 3. Discussion

This study showed that PLA2G1B secreted by the pancreas into the gastrointestinal tract contributes to inflammatory bowel disease, and its inactivation in mice protects against DSS-induced inflammation and colitis. The effects of PLA2G1B on colitis are similar to those observed with PLA2G2A secreted predominantly by macrophages, in which its inhibition was shown to protect rats from chemically induced colitis [17,35]. The effects of these secretory phospholipases are in striking contrast to those observed with another secretory phospholipase, PLA2G10, secreted by colonic epithelial cells, which was shown to suppress colitis, and its deficiency led to increased DSS-induced inflammation and colitis [15]. The protective effect of PLA2G10 was attributed to its preference for the selective mobilization of ω-3 polyunsaturated fatty acids from membrane phospholipids [15], a property that was not observed with PLA2G1B.

One shared property of phospholipase A_2_ enzymes including PLA2G1B, PL2G2A, and PLA2G10 is the hydrolysis of phospholipids at the *sn*-2 position to produce one fatty acid and a lysophospholipid. Whereas the protective effect of PLA2G10 was attributed to the liberation of ω-3 polyunsaturated fatty acids, the colitis-promoting effect of PLA2G2A was attributed to the liberation of ω-6 polyunsaturated fatty acids and the subsequent generation of eicosanoids in the colon after macrophage infiltration into the tissue in response to DSS-induced injury [35]. However, we did not find a difference in the LPC and LPA levels in the colons of the DSS-treated wild-type and *Pla2g1b^−/−^* mice, thus indicating that PLA2G1B plays a minimal role in phospholipid hydrolysis in the colon. It is likely that other PLA_2_ enzymes that are highly expressed in colonic epithelial cells, such as PLA2G12A, PLA2G2D, PLA2G5, PLA2G10, and PLA2G12B [36], are responsible for generating the bulk of LPC and LPA in the colons of DSS-treated mice. Importantly, these data indicate that the increased LPC and LPA levels in the colon is not sufficient to drive colitis after DSS treatment. Furthermore, unlike the effects of PLA2G2A and PLA2G10, which increase colitis via LPC and LPA elevation, the protective effects of PLA2G1B inactivation on DSS-induced inflammation and colitis are not due to alterations in phospholipid remodeling in the colon.

The protective effects of PLA2G1B inactivation were most evident during the recovery phase after the cessation of the DSS treatment. These observations suggest that PLA2G1B inactivation either accelerates intestinal barrier regeneration after injury and/or reduces inflammation in the colon to promote repair. Our histological data revealed increased numbers of stem and progenitor cells in the intestines of the *Pla2g1b^−/−^* mice compared to the wild-type mice. In view of reports showing that intestinal stem cell activation promotes intestinal epithelial regeneration [37,38], the reduced colitis disease index observed in the *Pla2g1b^−/−^* mice may be due, at least in part, to the increased stem cell reservoir in their intestines that can be activated during injury to accelerate epithelial regeneration. The relationship between PLA2G1B inactivation and accelerated epithelial repair was supported by observations of increased expressions of genes responsible for epithelial repair such as LRH-1, PPARγ, ZO-1, and CDH1 in the colons of DSS-treated *Pla2g1b^−/−^* mice compared to the wild-type mice.

It is important to note that although the negative impact of PLA2G1B on intestinal epithelial repair is reminiscent of the influence of PLA2G2A on intestinal stem cell proliferation, epithelial cell repair, and DSS-induced colitis [16], the mechanism by which PLA2G1B and PLA2G2A regulate intestinal stem cell proliferation and epithelial regeneration may be quite different. Whereas PLA2G2A is expressed in intestinal Paneth cells and influences stem cell niche through both intracellular YAP1 activation and extracellular effects to promote Wnt signaling and inflammation [16], PLA2G1B is not expressed in the intestine but is secreted from pancreatic acinar cells into the intestinal lumen in response to food intake to aid lipid nutrient absorption [39]. It is possible that the undigested phospholipids in the intestinal lumen are responsible for increased enterocyte proliferation and the observed epithelial regeneration in the *Pla2g1b^−/−^* mice. Consistent with this hypothesis is the previous report that showed that the biliary phospholipids secreted into the intestinal lumen promote enterocyte proliferation via the activation of the nuclear receptor, LRH1 [40,41]. Additionally, extracellular phospholipid hydrolysis has been shown to be required for cholesterol uptake into intestinal cells [42]. The reduced cholesterol uptake may lead to a compensatory increase in endogenous cholesterol biosynthesis via the sterol regulatory element-binding protein (SREBP)-mediated pathway, a process that has also been linked to intestinal stem cell proliferation [43]. Whether PLA2G1B deficiency promotes stem cell proliferation and epithelial repair exclusively through LRH1 or if SREBP-mediated pathways are also involved will require additional comprehensive studies.

The current study also revealed reduced inflammatory cytokine production in the colons of the DSS-treated *Pla2g1b^−/−^* mice compared to the wild-type mice. The reduced colonic inflammation cannot be due entirely to the accelerated epithelial regeneration in the intestines of *Pla2g1b^−/−^* mice because comparable expression levels of CD68 and EMR1 indicated that a similar number of macrophages was present in the colons of both groups. Hence, the reduced expression of inflammatory cytokines in the *Pla2g1b^−/−^* mice suggested that PLA2G1B may have a direct influence on the macrophage inflammatory response. Our in vitro experiments using GM-CSF-derived myeloid cells that mimic the role of GM-CSF in macrophage defense and wound healing in the intestine [44], and M-CSF-differentiated bone marrow cells that specifically produce macrophages, revealed that exogenous PLA2G1B directly activates macrophages to secrete proinflammatory cytokines. It is likely that PLA2G1B activates proinflammatory cytokine secretion via these macrophages by binding to M-type phospholipase A_2_ receptors and the activation of signal transduction pathways similar to that observed in human lung macrophages [45]. These studies identified a direct contributory role of PLA2G1B in DSS-induced colonic inflammation. Thus, PLA2G1B inactivation may also suppress DSS-induced colitis by reducing inflammation in the colon. These results suggest that specific PLA2G1B inhibition may be a viable option for the treatment of acute colitis as well as other inflammatory diseases.

## 4. Materials and Methods

### 4.1. Animals

Wild-type C57BL/6J mice purchased from Jackson Laboratories (Bar Harbor, ME, USA) were used to establish a breeding colony in our institutional facility. The *Pla2g1b^−/−^* mice were generated in our laboratory and backcrossed with C57BL/6J mice for >10 generations to generate *Pla2g1b^−/−^* mice in C57BL/6J background, as described in [18]. The C57BL/6J and *Pla2g1b^−/−^* mouse colonies were maintained in a temperature- and humidity-controlled room with a 12 h light/dark cycle. The animals were fed rodent chow (#5053; Purina Pico Labs, Cincinnati, OH, USA) with free water access. All experiments were performed according to experimental protocols approved by the institutional animal care and use committee at the University of Cincinnati in accordance with the *Guide for the Care and Use of Laboratory Animals* published by the US National Institutes of Health.

### 4.2. DSS-Induced Colitis

Male mice were given water with or without 3% (*w*/*v*) of 36k–50k molecular weight DSS (MP Biomedicals, Santa Ana, CA, USA) for 5 days followed by regular water without DSS for another 5 days, similar to the established protocol used to examine colitis in mice [46]. Body weights were measured daily. Stool consistency and the presence of occult blood was determined daily using the Sure-Vue^TM^ Fecal Occult Blood Slide Test System (23-038031, ThermoFisher, Cincinnati, OH, USA). Each parameter was assigned a score to determine the disease activity index (Table 1).

### 4.3. Colon Histology

Distal colon tissues were fixed in 10% formalin, processed, embedded in paraffin, and stained with hematoxylin and eosin. Images were taken using an Olympus BX61 microscope. Pathological score was determined based on crypt damage, depth of injury, and severity of inflammatory cell infiltration. The score was multiplied by a factor reflecting the percentage of affected area and added to obtain a total score (Table 2).

### 4.4. Colonic Lysophosphatidylcholine (LPC) and Lysophosphatidic Acid (LPA) Measurements

Colonic LPC and LPA contents were determined by flushing the isolated colons with phosphate-buffered saline and then flash-frozen at −80 °C. The colon tissues were thawed in buffer containing 50 mM Tris-Cl, pH 7.4, 150 mM NaCl, and 10 mM EDTA. The samples were extracted three times with n-butanol-saturated water, as described in [47]. The butanol fractions were dried under N_2_ stream and then rehydrated for determination of LPC and LPA levels with commercially available ELISA assay kits (MyBioSource, San Diego, CA, USA; #MBS2031889 and #MBS2024826, respectively).

### 4.5. Intestinal Stem and Progenitor Cell Identification

Intestine samples were fixed overnight in 10% neutral-buffered formalin and then embedded in paraffin for preparation of thin sections. The thin section slides were incubated with primary antibodies, which were rabbit anti-Olfm4 (1:500 dilution; Cell Signaling, Danvers, MA, USA; #39141) or rabbit anti-Id1 (1:200; CalBioreagents, San Mateo, CA, USA; #M082), to identify intestinal stem and progenitor cells, and then visualized using a VectaStain Elite ABC kit (Vector Labs, Newark, CA, USA; PK-6101). The numbers of stem and progenitor cells were assessed from ~100 crypts (N = 8 mice per group), as described in [43].

### 4.6. RNA Expression in Colon and Bone Marrow-Derived Myeloid Cells

RNA was isolated from the colons of DSS-treated mice or bone marrow-derived myeloid cells using TRIzol reagent (Invitrogen, Carlsbad, CA, USA;) and Direct-zol RNA miniprep kit (Zymo Research, Irvine, CA, USA;). RNA extracted from colons was also subjected to lithium chloride precipitation to remove polysaccharides. cDNA was synthesized from the extracted RNA using the qScript cDNA Synthesis Kit (QuantaBio, Beverley, MA, USA). Quantitative real-time PCR (RT-PCR) was performed with a StepOnePlus Fast thermocycler using Fast SYBR Green Master Mix (Applied Biosystems, Carlsbad, CA, USA). Sequence-specific primers are listed in Table 3. Expression levels of mRNA were normalized to cyclophilin using the ΔΔCT analysis method.

### 4.7. Plasma Cytokine Measurement

Blood was collected from DSS-treated mice after a 4 h fast for plasma preparation. Inflammatory cytokines in the plasma were measured using commercially available ELISA kits for CXCL1 (R&D Systems, Minneapolis, MN, USA; DY453-05), MIP-2 (ThermoFisher, Cincinnati, OH, USA; EMCXCL2), IL-1β (ThermoFisher, BMS6002), and IL-6 (R&D Systems, M6000B).

### 4.8. PLA2G1B-Induced Cytokine Expression In Vitro

Bone marrow cells isolated from tibias and femurs of wild-type C57BL/6J mice were flushed with Hanks’ balanced salt solution, subjected to red blood cell lysis (eBioscience, San Diego, CA, USA; 00-4300-54) for 5 min at room temperature, and passed through a 40 µm cell strainer. The isolated cells were cultured in the presence of 20 ng/mL GM-CSF (Peprotech, Cranbury, NJ, USA; 315-03) or 20% L929-conditioned media as source of M-CSF for 7 days to induce differentiation of monocytes into mature myeloid cells with dendritic cell and macrophage characteristics. The mature myeloid cells were then incubated with or without 10 µg/mL bovine PLA2G1B (Sigma Aldrich, Burlington, MA, USA; P8913) for 3 h prior to RNA isolation. Expression levels of inflammatory cytokines were determined via RT-PCR as described above.

### 4.9. Statistical Analysis

All data were expressed as the mean ± SD. Statistical analysis was performed using Graphic Prism version 5 software (GraphPad Software, Boston, MA, USA). Normality was examined using the Shapiro–Wilk test. All data showed parametric distribution and were evaluated for differences using Student’s *t*-test. Differences at *p* < 0.05 were considered statistically significant.

## 5. Conclusions

This study shows that PLA2G1B inactivation reduces DSS-induced colitis in mice by increasing the stem and progenitor cell populations in the intestine to accelerate intestinal epithelial repair as well as by reducing the proinflammatory responses of myeloid cells in the colon. Although these results may be interpreted to suggest that PLA2G1B-specific inhibition is a potential therapeutic option for inflammatory bowel disease management, a limitation of this study is that the study was conducted in mouse models. The translation of these results to a clinical setting requires direct preclinical experiments in humans. Moreover, whether increased stem and progenitor cell populations in the intestine with PLA2G1B inactivation may also increase cancer risk also needs to be addressed.

## Figures and Tables

**Table 1:** Disease activity index.

Score	Weight Loss	Stool Consistency	Blood in Stool
0	None	Normal pellets	None
1	1–5%	Slightly loose but shaped	Hemoccult positive
2	5–10%	Loose pellets	Visible slight bleeding
3	10–15%	Loose feces and no shape	Obvious bleeding, no adhesion around anus
4	>15%	Diarrhea	Gross bleeding, blood encrustation around anus

**Table 2:** Colon histology score.

Histology Grade		Area		Immune Cell Infiltration	
0	Normal morphology	×1	0–25%	0	None
1	<1/3 crypt damage	×2	26–50%	1	Crypt base
2	Loss of crypt	×3	51–75%	2	Mucosa
3	Crypt and epithelium loss	×4	76–100%	3	Submucosa

**Table 3:** Primers used for qPCR.

Name	Forward Primer	Reverse Primer
Cyclophilin	TCATGTGCCAGGGTGGTGAC	CCATTCAGTCTTGGCAGTGC
CD68	TTTCTCCAGCTGTTCACCTTGA	CCCGAAGTGTCCCTTGTCA
EMR1	TGTCTGACAATTGGGATCTGCCCT	ATACGTTCCGAGAGTGTTGTGGCA
MCP-1	CTTCCTCCACCACCATGCA	CCAGCCGGCAACTGTGA
MIP-1α	TTTGAAACCAGCAGCCTTTGCTCC	TCAGGCATTCAGTTCCAGGTCAGT
MIP-2	CCCTCAACGGAAGAACCAAA	AGGCACATCAGGTACGATCCA
CXCL1	TGGCTGGGATTCACCTCAAGAACA	TGTGGCTATGACTTCGGTTTGGGT
IL-1β	CTACAGGCTCCGAGATGAACAAC	TCCATTGAGGTGGAGAGCTTTC
IL-6	CCGGGAACGAAAGAGAAGCT	GCGCTTGTGGAGAAGGAGTT
TNFα	ATCCGCGACGTGGAACTG	ACCGCCTGGAGTTCTGGAA
PPARγ	CTGCAGGCCCTGGAACTG	CGATCTGCCTGAGGTCTGTCA
LRH-1	TCACATCTCCCATTAGCATGACA	GGAAAGTGACCATAGGGTTGGTAA
ZO-1	CCTCCGTTGCCCTCACAGTA	GGGCGCCCTTGGAATG
CDH1	TGTGGGTCAGGAAATCACATCTT	CCGATACGTGATCTTCTGATCCA

## Data Availability

The data supporting this study are available within the article and from the corresponding author (David Y. Hui, huidy@ucmail.uc.edu) upon request.
